# Potential Regulatory Role of Gibberellic and Humic Acids in Sprouting of *Chlorophytum borivilianum* Tubers

**DOI:** 10.1155/2014/168950

**Published:** 2014-02-13

**Authors:** Jaafar Juju Nakasha, Uma Rani Sinniah, Adam Puteh, Siti Aishah Hassan

**Affiliations:** Department of Crop Science, Faculty of Agriculture, Universiti Putra Malaysia, 43400 Serdang, Selangor, Malaysia

## Abstract

Tubers of safed musli (*Chlorophytum borivilianum*) were immersed in three different concentrations of gibberellic acid (GA_3_) or humic acid (HA) prior to planting. The highest concentration of GA_3_ (20 mg L^−1^) and all concentrations of HA (5, 10, and 15%) appeared to hasten tuber sprouting and promote uniform sprouting pattern. The use of 20 mg L^−1^ GA_3_ or 15% HA successfully improved sprouting and mean sprouting time. Safed musli growth and development was improved through the increase in the number of leaves, total leaf area, leaf area index, and total fibrous root length. This directly influenced the number of new tubers formed. The use of 20 mg L^−1^ GA_3_ or 15% HA gave similar response with nonsignificant difference among them. However, due to the cost of production, the result from this study suggests that 15% HA should be used to obtain improved sprouting percentage, homogeneous stand establishment, efficient plant growth and development, and increased yield of safed musli.

## 1. Introduction 

Safed musli, botanically known as *Chlorophytum borivilianum*, is an herb which originated from India. This herb has been referred as a wonder herb due to its usage in many Ayurvedic medicines for strength and vigour particularly in overcoming impotence. The tuberous roots also termed as “golden roots” contain saponins (17%), more than 25 alkaloids, calcium, and protein constituents [1]. The roots of safed musli which contain aphrodisiac properties have also been scientifically proven to have antistress and antioxidant properties and [2] hence its potential in the herbal industry. Tubers of safed musli often serve a dual purpose, namely, as organ of economic importance for extraction of saponins as well as the organ used as planting materials. Tubers are preferred as planting material because the seeds are highly dormant resulting in low germination [3]. In addition, plants grown from seeds require two to three years for tuber maturation to occur as it is a biannual plant. In India, tuber sprouting has been reported to occur only during the rainy season, being dormant otherwise [4]. Although dormancy may be advantageous in selected cases, for example, it allows the product to stay in the market for a longer period, as the main source of input for cultivation, it is generally an undesirable characteristic, where rapid, uniform stand establishment and growth are required. In order to overcome dormancy, farmers normally have to wait for the planting material to naturally end its dormancy or subject to dormancy breaking treatment, before it is used for planting.

In the year 2006, safed musli was introduced into Malaysia. Due to differences in agroclimatic condition, it was found that safed musli germinated all year round in Malaysia and was not confounded to rainy season only. However, sprouting was variable requiring two weeks to two months resulting in heterogeneous stand establishment which complicates the fertilization and irrigation schedule. In addition, it disrupts the farm management practices such as deflowering and harvesting. Hence there is a need to establish a protocol to obtain more rapid and synchronized stand establishment for safed musli. Often, dormancy breaking prior to planting is practiced to enhance sprouting, especially to obtain rapid and uniform sprouting. It appears that the use of growth regulator is potentially one of the most suitable methods to enhance germination in many crops [5, 6].

According to Tanno et al. [7], dormancy in tuberous crop is caused by physiological factor, where the amount of endogenous hormone is not sufficient to promote sprouting. Therefore, the addition of growth regulator may raise the endogenous hormone level and break tuber dormancy. Gibberellic acid and auxin have been implicated to play a role in stimulating bud emergence [5, 8]. Positive response of gibberellic acid on enhancement of sprouting was reported by Choudhuri and Ghose [9], in potato, but these authors also stated that concentration plays an important role as increased concentration of 25 to 100 mg L^−1^ caused deformation of tubers. Slomnicki and Rylski [10] on the other hand found that the use of lower concentration of gibberellic acid at 5 to 40 mg L^−1^ was effective. Hence the need to carry out systematic study on type and concentration of the phytohormone is used to ensure that exogenous application of growth regulator does not bring harm to the plant growth and development at the later stage.

Another growth regulator that is closely related to the function of gibberellic acid is auxin. Auxin plays an important role in initiating buds in many plants, a role similar to gibberellic acid. However, in recent years, humic acid which mimics the function of auxin [11] has become relatively popular and many reports on the advantageous of humic acid are available [6, 12]. It is used as a substitute to auxin in order to minimize the cost of production. However, reports on the use of humic acid to enhance sprouting are scarce, though positive results have been reported in improving plant growth and development [13]. To date there is no published report on dormancy breaking of safed musli tubers, although several reports on other tubers are present. Hence the objective of this study was to test the effectiveness of gibberellic acid (growth regulator) and humic acid (growth regulators-like), with three different concentrations each, on the ability to break dormancy, improve sprouting, obtain uniform field stand, and to study the effect on yield of safed musli.

## 2. Materials and Methods 

The study was conducted at Universiti Putra Malaysia in the year 2011. Whole tubers with a fresh weight of 9 ± 2 g each were selected as planting material. Tubers were subjected to treatment with either gibberellic acid (GA_3_) or humic acid (HA). Three concentrations of GA_3_ were used, namely, 10, 15, and 20 mg L^−1^ while for HA commercial HA (brand: Humac Solu^n^ 50, Thailand) derived from leonardite was used at concentrations of 5, 10, and 15%. Tubers were soaked for 1 h in different concentrations of GA_3_ and HA and distilled water was used as the control. After 1 h, a total of 420 tubers were air-dried under shade for 15 min and were then sown in polybags measuring 36 × 41 cm containing river sand and top soil at a ratio of 2 : 1. Each treatment was replicated four times with 20 plants per replicate. The plants were fertilized with diammonium phosphate (DAP, 46.2% P_2_O_5_) at a rate of 100 kg per hectare at 45 days after planting. The application of DAP at this stage is essential to support the initiation, growth of the specialized roots, and also the filling process. This was followed by urea (46% N) at 50 kg per hectare at 75 days after planting while muriate of potash (MOP, 60% K_2_O) at the rate of 50 kg per hectare was applied at 105 days after planting.

Tubers were recorded as “sprouted” when the sprout length was 2 mm above soil level and sprouting was recorded daily until no further sprouting was observed. Sprouting percentage was computed and reported for 10, 20, 30, 40, 50, and 60 days after planting (DAP). Mean sprouting time was calculated according to the equation [14]

(1) $$\begin{array}{c} \text{mean sprouting time (MST)}=\frac{\Sigma \left(\text{D}n\right)}{\Sigma n}, \end{array}$$

where *n* is the number of tubers, which sprouted on day D and D is the number of days counted from the beginning of planting.

Shoots from each treatment which sprouted on the same day were tagged, and days after sprouting (DAS) was used for sampling in order to obtain shoot and root length during the early growth on weekly basis until 28 DAS. Subsequently plant development at 60, 90, and 120 DAS was recorded by obtaining data on number of leaves, total leaf area (using Leaf Area Meter 3100, USA), fibrous root length (using root image analyzer, Epson 1680, USA), and leaf area index (LAI). Leaf area index was calculated by dividing the total leaf area per plant into the ground area (canopy). Tuber diameter, length, and dry weight were collected on 120 DAS as this is the final stage before leaf senescence occurred. Yield was presented based on tuber dry weight per plant collected at 270 DAS. All parameters were collected based on 10 plants per replicate, with a total of four replicates for each treatment. Data obtained was subjected to analysis of variance using statistical analysis system (SAS) and treatment means were compared using least significant difference (LSD) test at 5% level of probability.

## 3. Results and Discussion

### 3.1. Sprouting Percentage and Mean Sprouting Time

Sprouting was significantly influenced by both GA_3_ and HA treatments (Table 1). Control tubers had the lowest sprouting; recording 17% at 10 DAP. High concentration of GA_3_ at 20 mg L^−1^ recorded three times higher sprouting (60%) at 10 DAP, while tubers treated with all concentrations of HA had higher sprouting percentage compared to GA_3_ treatment, especially those treated with 10% and 15% HA which showed more than 70% tuber sprouting at 10 DAP. The effect of using growth regulators to enhance synchronous sprouting appeared to be effective even at the very early stage of *circa* one week after sprouting.

Sprouting percentage computed at 20 DAP showed that control tubers had increased to 42% sprouting which is double that observed at 10 DAP. Both 10 and 15 mg L^−1^ GA_3_ treated tubers, which initially were slow in sprouting, were triggered to sprout with more than 75% sprouting at 20 DAP. Treatments which had high sprouting of more than 60% at 10 DAP (all HA treatments and 20 mg L^−1^ GA_3_) were able to increase sprouting to a maximum of around 85 to 91%. In most cases except for control and 10 mg L^−1^ GA_3_ no further increase in sprouting was obtained after 20 days of planting although data was collected up till 60 DAP. Data also showed that HA treatment not only hastened sprouting but also increased the sprouting percentage. This is in agreement with results reported for other crops such as potato [6] where the application of HA resulted in increased sprouting percentage. Similar response was also observed for wheat seeds where HA treated seeds had higher germination percentage compared to control [12]. Untreated tubers (control) showed a sequential increase in sprouting percentage from 17% at 10 days to 82% at 60 DAP, clearly showing heterogeneous sprouting pattern. The implication of heterogeneous sprouting is on the agronomic practices such as fertilization and deflowering which will affect the maturity and tuber bulking process. In addition to sprouting percentage, MST revealed that all concentrations of HA and 20 mg L^−1^ GA_3_ had lower MST whereby only eight days were required for most of the tubers to sprout (Table 1). Tubers treated with 10 and 15 mg L^−1^ GA_3_ on the other hand required more than two weeks for average number of tubers to undergo sprouting. The longest MST was found from control tubers, which is 25.8 days.

### 3.2. Sprout Length and Total Root Length during Early Growth

The effects of GA_3_, HA, and distilled water (control) treatments on the sprout length and root length are shown in Table 2. The sprout and root length were significantly affected by the application of GA_3_ and HA. All treated tubers had elongated sprout at seven DAS as compared to control with 10% HA having the longest sprout length (1.4 cm) and this was followed by 15% HA at 1.2 cm. Tubers treated with GA_3_ irrespective of concentrations had similar sprout length ranging from 0.60 to 0.65 cm. By 14 DAS, control tubers were still the lowest performer with mean sprout length of 0.73 cm. GA_3_ treated tubers had almost doubled in sprout length, and HA treated tubers also increased in sprout length but the increase as compared to seven DAS was lower.

At 21 DAS, an increase in sprout length was recorded for all treatments with 20 mg L^−1^ GA_3_ and all HA treated tubers stabilizing at a height of around 2.3 to 2.6 cm. Sprout elongation is usually determined by the amount of storage material present in the mother tubers as well as the degradation efficiency of starch to soluble sugars for energy supply to the developing sprout [15]. This conversion is dependent on the switch or signals which supposedly change from storage to sprouting related mode. Panneerselvam and Jaleel [16] showed that there was a slight decrease in starch breakdown during the initial sprout emergence, but, as the sprout continued to grow, a sharp decrease in starch was found in yam tubers and turmeric rhizomes.

**Figure 1:**
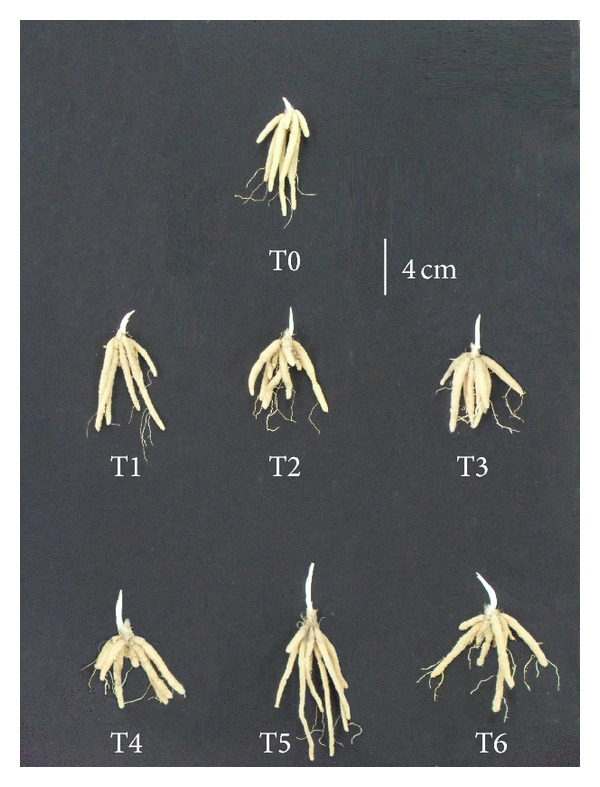
The elongation of sprout and root at 28 DAS as affected by different treatments. Tubers treated with T3 and T6 had the longest root length among all treatments. The longest sprout length was recorded from treatment T5. Note: T0 = control; T1 = 10 mg L^−1^ GA_3_; T2 = 15 mg L^−1^ GA_3_; T3 = 20 mg L^−1^ GA_3_; T4 = 5% HA; T5 = 10% HA; T6 = 15% HA.

By 28 DAS, tubers from control and 10 and 15 mg L^−1^ GA_3_ treatment still showed poor sprout growth, with a sprout length of less than 2.5 cm. However, increasing the concentration of GA_3_ to 20 mg L^−1^ gave significantly higher sprout length of 3.6 cm. In the case of tubers treated with HA, 10% HA had sprout length which was double (4.10 cm) compared to control 1.97 cm (Figure 1).

In the case of root development, the highest concentration of GA_3_ and HA showed good development of roots during the early stage (Table 2). These treatments were at least double than control on 7, 14, and 21 DAS. Near the end of the early growth stage (28 DAS), 20 mg L^−1^ GA_3_ and 15% HA superseded other treatments with 14 to 15 cm longer root length compared to control (Figure 1). Well-developed roots are crucial during early growth, as it will support the expansion of leaves in the next phase through nutrient and mineral absorption.

### 3.3. Number of Leaves

Data collection on sprout length ended at 28 DAS as some of the treatments began to show leaf expansion. Expanded leaves were counted at 60, 90, and 120 DAS and it was observed that tubers with longer sprout length had an advantage over tubers with shorter sprout length, as leaf expansion began earlier compared to those having shorter sprout length. Leaves expand to intercept light for photosynthesis and are the main source of CO_2_ assimilation in plants. Photosynthetic rates are generally low in young unexpanded leaves and increase when the leaves become fully expanded. Hence, early expansion will render the plants with ability to photosynthesize and coordinate the physiological processes within the plant. Tubers treated with 15 mg L^−1^ GA_3_, 20 mg L^−1^ GA_3_, and 15% HA produced 17 to 18 leaves per plant at 60 DAS (Table 3) while control had the lowest number of leaves [14]. The increase in number of leaves will equate to higher photosynthetic activity, thus providing higher photoassimilate for tuber bulking. Emergence of new leaves continued for all treatments even at 90 DAS. The increase in number of leaves is due to the great demand from the tubers as it is actively undergoing the filling process. Plants belonging to the control category consistently had lower number of leaves at all stages of plant growth and development. A unique characteristic of safed musli is in relation to the tuber cropping cycle which is about 8-9 months, but after 3–3.5 months of sowing the leaves start yellowing; subsequently they become dry and detached from the tuber. The tubers, at this stage, though being formed completely, are not mature enough to be harvested. The process termed hardening has to occur via the formation of cuticle before they are ready for harvest. Hence the presence of sufficient amount of foliage within the 3–3.5 months is of consequence as it will ensure proper tuber bulking. Consequently, a small change in number of leaves can cause a big difference in leaf area and leaf area index of the plant which is highly related to net primary productivity which subsequently influences tuber bulking. According to Khan and Khalil [17] the rate of dry matter accumulation is a function of leaf area of a crop, because the light interception is mainly associated with leaf area. Hence, these parameters are reported and discussed in the upcoming sections of the paper.

As the plants reached 120 DAS, some of the leaves had already dried (senescence phase) and detached themselves from the plants crown which resulted in a decrease in number of leaves. However, plants which had more number of leaves at 90 DAS had advantage over the plants which had less number of leaves as it sustained longer in the senescence phase before all the leaves dried completely. All concentrations of GA_3_, as well as two concentrations from HA (5% HA and 15% HA), had 16 to 18 leaves per plants, whereas others had only 13 to 14 leaves.

### 3.4. Total Leaf Area

The elongation of sprout and root at the earlier stage affected the leaf area at the later stage of plant growth and development. It was observed that plants with longer sprout were able to expand leaves faster compared to plants having slow sprout growth at the initial stage. At 60 DAS, increasing GA_3_ concentration increased total leaf area. Total leaf area for 20 mg L^−1^ GA_3_ treatment was 1.6 times higher compared to control (Table 3). The difference in total leaf area not only was due to contribution by the number of leaves but also was influenced by the size of each leaf in the plant. As the total leaf area and number of leaves were similar for 20 mg L^−1^ GA_3_ and 15% HA treatment at 60 DAS, namely, 17 and 18 leaves, respectively, it means that the size of each leaf in both treatments was also similar. Both treatments continued to show the highest total leaf area among all treatments which was 792 cm^2^ for 20 mg L^−1^ GA_3_ and 751 cm^2^ for 15% HA at 90 DAS. The total leaf area for all other treatments ranged from 493 to 633 cm^2^ with the lowest being for treatment with 10% HA. By 120 DAS, there was a decrease in total leaf area for all treatments as some of the leaves had started to wither. All treatments showed at least 50% reduction of total leaf area from 90 to 120 DAS with the highest reduction percentage from control (61%).

### 3.5. Leaf Area Index

Due to increase in number of leaves and concomitant increase in total leaf area in some treatments, it was observed that increasing the concentration of both growth regulators led to an increase in leaf area index (Table 4). At 60 DAS, the lowest leaf area index was noticed from control plants (0.44), which had lower number of leaves with smaller leaf area as compared to other treatments. However, minimal difference was recorded between control, low concentration of GA_3_ (10 and 15 mg L^−1^ GA_3_), and low concentration of HA (5 and 10%) where the leaf area index ranged from 0.4 to 0.58. The plant from treatment with the highest concentration of both GA_3_ and HA had high leaf area index of around 0.7. Leaf area index values over 1 indicate a layered canopy with multiple layers of leaves per unit ground surface area. The value for leaf area index obtained for safed musli in this study is relatively low but is in agreement with report by Somanath [18] for safed musli planted in India and appear to fall within the published average LAI value for grassland species which is in the range of 0.3 to 2.0 [19].

The leaf area index continued to increase parallel to the increase in number of leaves and expansion at 90 DAS. The value obtained from control on 90 DAS is similar with what was obtained from 20 mg L^−1^ GA_3_ and 15% HA at 60 DAS. Both 20 mg L^−1^ GA_3_ and 15% HA had increased leaf area index to 1.09 and 1.03, respectively. As the plants undergo senescence at 120 DAS, the leaf area index had decreased by more than 50% for all treatments especially in control. Control plants had 61% decreases in leaf area index value, indicating faster senescence compared to other treatments. Although senescence had progressed in all treatments, plants from treatments 20 mg L^−1^ GA_3_ and 15% HA retained relatively high leaf area index of 0.53 and 0.46, respectively.

### 3.6. Total Fibrous Root Length

Since there was a difference in root length at the earlier phase, total root length at the later stage was also affected. The total fibrous root length differed considerably between treatments and ranged from 980 to 2453 cm at 60 DAS depending on treatments (Table 5). Tubers treated with GA_3_ showed that increasing the concentration of GA_3_ resulted in increased total fibrous root length, with 15 and 20 mg L^−1^ GA_3_ having similar total fibrous root length, 2236 and 2453 cm, respectively. The fibrous root length for these treatments was at least 2.3 times longer compared to control which had 980 cm total root length at 60 DAS.

**Figure 2:**
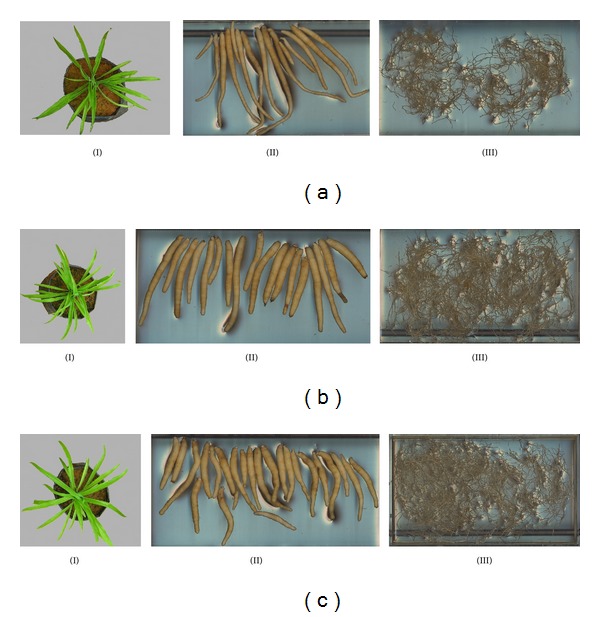
Images of leaves (I), tuberous roots (II), and fibrous roots (III) of safed musli at 90 DAS with (a) for control, (b) for tubers treated with 20 mg L^−1^ GA_3_, and (c) for tubers treated with 15% HA. Tuberous roots from control (a)(II) were less in number and were elongated while tubers treated with 15% HA (c)(II) were significantly higher in number with shorter tuber length. Similarly treated tubers had higher number of leaves (b)(I) and (c)(I) and total fibrous root length (b)(III) and (c)(III) compared to control (a)(I) and (a)(III).

By 90 DAS, similar pattern was observed where two higher concentrations of GA_3_ (15 and 20 mg L^−1^ GA_3_) were not significantly different with each other with both having 2698 cm and 2928 cm, respectively. Most of the treatments showed a gradual increase in total fibrous root length, while tubers treated with 15% HA showed a remarkable increase in fibrous root length from 2077 cm at 60 days to 3441 cm at 90 DAS, having the longest total fibrous root length (Table 5 and Figure 2). Despite the difference in total fibrous roots length at 90 DAS, all treatments showed no significant difference at 120 DAS, *albeit* a lower value for total fibrous roots length. A sharp reduction in this parameter was recorded in 15% HA treatment where it decreased from 3441 cm at 90 DAS to 1092 cm at 120 DAS, while other treatments only showed gradual reduction. The reduction in fibrous roots coincided with the leaf senescence. As all the leaves began to undergo senescence above ground, similar event also occurred underground where the fibrous roots detached from the tubers leaving only very minimum length (less than 20 cm total fibrous root length for each tuber bulk).

### 3.7. Tuber Dry Weight

Differences, observed during the vegetative growth of safed musli as affected by the treatments, especially in relation to leaf area index, are expected to influence tuber dry weight. There was a significant difference between treatments on tuber dry weight (Table 5). Both GA_3_ and HA showed the same trend whereby increasing the concentration resulted in increased tuber dry weight, with an exception for 10% HA probably due to the poor initial development during the early growth stage.

At 60 DAS, control and 10% HA treated tubers were actually about 3.5 times lower compared to dry weight of tubers treated with the highest concentration of GA_3_ and HA (4.5 g and 4.1 g, resp.). Interestingly, at 90 DAS, plants from 15% HA treatment increased in tuber dry weight and become 1.8 g heavier compared to 20 mg L^−1^ GA_3_ treatment, despite showing the same values for tuber dry weight at 60 DAS. This could be attributed to the well-developed fibrous roots from 15% HA treatment which may have contributed to better nutrient and mineral uptake. Furthermore, it was found that the lowest concentration of HA produced higher tuber dry weight (5.2 g) compared to the lowest concentration of GA_3_ (3.8 g)_._ There was an increase in tuber dry weight for all treatments at 120 DAS. Although at 120 DAS the plant had begun to enter senescence phase, active tuber filling was noted. The increase in accumulation of dry matter during the senescence phase has also been reported in potato [20]. There was an increase in tuber dry weight for control plants from 3.4 g (at 90 DAS) to 5.3 g at 120 DAS. Meanwhile, the highest concentration of GA_3_ had 10.9 g of tuber dry weight, and this was not significantly different with the highest concentration of HA (11.5 g).

Changes in tuber dry weight are an important measurement in order to understand the influence of leaves and fibrous root development on dry matter production and on tuber bulking. More precisely, previous authors stated that knowledge of tuber dry matter production during the vegetative growth will help in making prediction and estimation of the potential yield [20]. On top of this, other characteristics are also important, as the dry weight alone is not sufficient to know the morphological characteristics of the tubers. Particularly, tuber dry weight data cannot provide information on whether the bulk of tubers produced are those with relatively few tubers but bigger in diameter or vice versa. In this respect, morphological characteristics which correspond to the tuber dry weight are discussed in the following subtopic. In tuberous crops such as safed musli harvesting can pose a major problem as tubers which are deeply embedded in the soil may break during harvesting. Thus, increased number of tubers with reduced length and increased diameter would be ideal to allow efficient harvesting.

### 3.8. Number of Tubers, Tuber Length, and Tuber Diameter

Treatments prior to planting which affected the sprouting pattern, development of leaves, and fibrous roots also influenced the morphological characteristics of the new tubers. Data on number of tubers, length, and diameter were collected only on 120 DAS as tuber growth and development cease at this point. Further changes are in relation to biochemical constituents and tuber color due to formation of cuticle layer.

Number of tubers recorded in this study ranged from 9.7 to 21.5 per bulk (Table 6). The lowest number of tubers produced was from 10% HA treatment. This was followed by control, 10 mg L^−1^ GA_3_, and 5% HA which produced 12 to 14 new tubers. The above-mentioned treatments plus 10% HA seem to have only minor difference with the original number of tubers that were used as the planting material (nine to 12 tubers per bulk). Hence, these treatments were not beneficial in increasing the number of tubers. In contrast, treatments with 20 mg L^−1^ GA_3_ and 15% HA produced double the number and recorded the highest number of tubers (19.7 and 21.5 tubers, resp.).

In addition, the treatments not only affected the number of tubers that were produced but they also affected the average tuber length. The tuber length is an important feature in safed musli as this will implicate the total yield upon harvest. It is not practical to have long tubers, as the tubers may break and remain below ground without being noticed during the harvesting. Eventually, there will be a lost in the total yield. In this study, it was found that increasing the concentration of GA_3_ and HA resulted in decreased tuber length (Table 6). Tubers from control, 10 mg L^−1^ GA_3_, 5% HA, and 10% HA had longer tubers of more than 12 cm. However, treatment with 15 mg L^−1^ GA_3_, 20 mg L^−1^ GA_3_, and 15% HA appeared to produce shorter tubers of 9.18 cm, 8.54 cm, and 9.83 cm, respectively. Based on the data from tuber length and number of tubers, it can be seen that the more number of tubers produced is, the lesser the tuber length is.

Treatments prior to planting with either GA_3_ or HA had no effect on the tuber diameter, even though it affected number of tubers and average tuber length. Although tubers treated with 15% HA had the biggest diameter (5.8 mm), this was not statistically different compared to control which had slightly smaller tuber diameter (5.1 mm). The differences observed for tuber morphological characteristics proved that although the treatments influenced leaves and fibrous root growth, it only affected the number of tubers and tuber length but not the diameter of the tubers produced. Despite some treatments having higher photoassimilate production compared to others, the photoassimilate was channeled towards increasing number of tubers and length. The final tuber filling processes occurred at the same rate with plants having lower photoassimilate production, as reflected by similar tuber diameters in all treatments.

### 3.9. Yield

**Figure 3:**
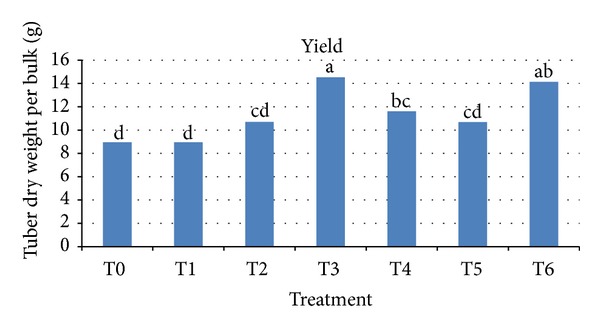
Final tuber dry weight (g) per bulk for each treatment. Note: T0 = control, T1 = 10 mg L^−1^ GA_3_, T2 = 15 mg L^−1^ GA_3_, T3 = 20 mg L^−1^ GA_3_, T4 = 5% HA, T5 = 10% HA, and T6 = 15% HA.

Tubers were harvested at 270 DAS to determine the yield. Both GA_3_ and HA showed increase in tuber dry weight corresponding to increase in concentration. However, it is assumed that the effect of treatment prior to planting by GA_3_ and HA may not directly affect the yield, but it influenced yield by causing changes during early growth phase such as increased speed of leaf expansion. Control, 10 mg L^−1^ GA_3_, 15 mg L^−1^ GA_3_, and 10% HA treated tubers were not significantly different with all having similar tuber dry weight of 9 to 11 g (Figure 3), while the highest concentration of GA_3_ and HA appeared to have superior effect where both resulted in increased tuber dry weight of more than 14 g per bulk, equivalent to more than 70 g fresh weight (an increase of 8 folds compared to the initial planting material used). Enhancement of grain yield production up to 8% through application of plant growth regulator has also been published in a recent past report with rice [21].

Based on the data collected, presented, and discussed above, two treatments showed strong positive effects in enhancing sprouting, minimizing time to sprouting, and having increased yield. Thus, comparison on increase in percentage of yield and cost of 20 mg L^−1^ GA_3_ and 15% HA was made (Table 7). It shows that soaking the tubers with 20 mg L^−1^ GA_3_ had increased tuber weight per bulk by 38% compared to tuber weight from control, while 15% HA had 37% increase in tuber weight. In order to observe the total increase in yield per ha, calculation on tuber dry weight per bulk together with the sprouting percentage on one ha of land was done. It was found that tubers treated with 15% HA had higher percentage of yield per ha compared to 20 mg L^−1^ GA_3_ (17% and 19%, resp.). This is due to the higher sprouting percentage in tubers treated with 15% HA. Consequently, basic cost analysis showed that treatment with 15% HA increased the cost of production by 2%, whereby the treatment with 20 mg L^−1^ GA_3_ showed an increase up to 8% which is four times higher, compared to 15% HA.

## 4. Conclusions

The ability of exogenous application of growth regulators to promote homogeneous sprouting has been proven successful in this study. Sprouting occurred earlier in all treated tubers as compared to control, with those being treated with 20 mg L^−1^ GA_3,_ 15% HA, and 20% HA particularly having high sprouting percentage even as early as 10 DAP. This is supported strongly by the data on MST which gave a value of 8 days for treatment with all concentrations of HA and 20 mg L^−1^ GA_3_, compared to 26 days for control. It is important to note that untreated control continued to sprout up to 60 DAS which contributed towards heterogeneous stand establishment. The early sprouting induced sprout elongation which at a later stage allowed efficient leaf expansion improved photosynthetic rate and effective tuber bulking. The improvements observed in shoot characteristics were also seen in root characteristics which combined to provide better growth and development in safed musli. Tubers treated with 20 mg L^−1^ GA_3_ and 15% HA had similar influence and showed higher yield compared to other treatments. However, based on the cost and benefit analysis, it is recommended to use 15% HA as the treatment to enhance safed musli tuber sprouting. The ability of HA to increase sprouting percentage within a short time will promote homogenous sprouting in safed musli, help to improve the agronomic management practices as the plants are uniform, and will reach harvest maturity simultaneously. In addition, the use of HA (15%) has proven to increase the yield by 37% with a mere 2% increase in the cost of production.

## Figures and Tables

**Table 1:** Cumulative sprouting percentage (%) and mean sprouting time (days) of safed musli tubers as affected by different concentrations of gibberellic acid (GA_3_) and humic acid (HA).

Treatments	Days after planting						MST (days)
	10	20	30	40	50	60	
Control	16.6^e^	41.5^d^	57.4^c^	65.6^c^	76.1^c^	81.9^c^	25.8^a^
10 mg L^−1^ GA_3_	21.9^d^	77.9^c^	88.1^ab^	88.1^ab^	88.1^ab^	88.1^ab^	15.8^b^
15 mg L^−1^ GA_3_	20.5^d^	87.8^ab^	87.8^ab^	87.8^ab^	87.8^ab^	87.8^ab^	14.3^b^
20 mg L^−1^ GA_3_	60.2^c^	89.1^ab^	89.1^ab^	89.1^ab^	89.1^ab^	89.1^ab^	8.1^c^
5% HA	64.3^b^	85.6^b^	85.6^b^	85.6^b^	85.6^b^	85.6^bc^	8.1^c^
10% HA	75.8^a^	89.2^ab^	89.2^ab^	89.2^ab^	89.2^ab^	89.2^ab^	8.6^c^
15% HA	78.0^a^	91.3^a^	91.3^a^	91.3^a^	91.3^a^	91.3^a^	7.3^c^

Prob. > *F*	3.31	3.79	5.18	4.74	4.79	5.31	5.02

MST indicated mean sprouting time. Means with the same letter in the same column are not significantly different at *P* < 0.05 by LSD.

**Table 2:** Sprout length (cm) and total root length (cm) during early growth as affected by concentration of gibberellic acid (GA_3_) and humic acid (HA).

Treatments	7 DAS		14 DAS		21 DAS		28 DAS	
	SL	TRL	SL	TRL	SL	TRL	SL	TRL
Control	0.48^e^	2.53^d^	0.73^e^	3.90^c^	1.23^c^	9.23^d^	1.97^d^	21.55^c^
10 mg L^−1^ GA_3_	0.65^d^	3.23^bc^	1.45^bc^	7.73^ab^	2.00^b^	13.28^bc^	2.40^c^	26.53^bc^
15 mg L^−1^ GA_3_	0.63^d^	3.48^bc^	1.32^c^	7.05^b^	1.53^c^	14.23^b^	2.28^cd^	28.85^b^
20 mg L^−1^ GA_3_	0.60^d^	4.30^a^	1.70^a^	8.95^a^	2.55^a^	21.88^a^	3.55^b^	36.40^a^
5% HA	0.85^c^	3.03^cd^	1.35^cd^	5.15^c^	2.31^ab^	14.83^b^	3.28^b^	29.93^b^
10% HA	1.35^a^	0.00^e^	1.75^a^	4.08^c^	2.55^a^	11.18^cd^	4.10^a^	24.80^bc^
15% HA	1.18^b^	3.85^ab^	1.50^b^	7.93^ab^	2.43^a^	20.83^a^	3.43^b^	35.20^a^

Prob. > *F*	0.16	0.77	0.13	1.49	0.25	2.91	0.39	5.27

DAS, SL, and TRL indicate days after sprouting, sprout length (cm), and total root length (cm), respectively. Means with the same letter in the same column are not significantly different by LSD.

**Table 3:** Number of leaves and total leaf area (cm^2^) per plant as affected by different concentrations of gibberellic acid (GA_3_) and humic acid (HA).

Treatments	60 DAS		90 DAS		120 DAS	
	NL	TLA	NL	TLA	NL	TLA
Control	13.80^cd^	321.50^c^	15.85^c^	504.35^cd^	12.85^c^	198.35^d^
10 mg L^−1^ GA_3_	14.89^bcd^	355.75^bc^	18.90^b^	594.96^b^	16.48^ab^	275.12^bcd^
15 mg L^−1^ GA_3_	16.72^abc^	423.25^ab^	18.60^b^	633.38^b^	15.73^abc^	287.58^bc^
20 mg L^−1^ GA_3_	17.34^ab^	504.25^a^	21.88^a^	792.05^a^	17.47^ab^	388.98^a^
5% HA	15.38^abcd^	362.25^bc^	18.64^b^	579.33^bc^	15.64^abc^	258.02^cd^
10% HA	13.04^d^	294.25^c^	17.30^bc^	492.95^d^	14.34^bc^	223.85^cd^
15% HA	18.37^a^	482.50^a^	21.30^a^	750.83^a^	18.37^a^	342.41^ab^

Prob. > *F*	3.39	97.99	2.29	86.11	3.31	79.39

DAS, NL, and TLA indicate days after sprouting, number of leaves, and total leaf area (cm^2^), respectively. Means with the same letter in the same column are not significantly different by LSD.

**Table 4:** Leaf area index per plant as affected by different concentrations of gibberellic acid (GA_3_) and humic acid (HA).

Treatments	Leaf area index		
	60 DAS	90 DAS	120 DAS
Control	0.44^c^	0.70^c^	0.27^c^
10 mg L^−1^ GA_3_	0.49^bc^	0.81^c^	0.37^bc^
15 mg L^−1^ GA_3_	0.58^abc^	0.87^bc^	0.40^abc^
20 mg L^−1^ GA_3_	0.69^a^	1.09^a^	0.53^a^
5% HA	0.50^bc^	0.79^c^	0.35^bc^
10% HA	0.40^c^	0.68^c^	0.31^c^
15% HA	0.66^ab^	1.03^ab^	0.46^ab^

Prob. > F	0.18	0.21	0.14

DAS indicates days after sprouting. Means with the same letter in the same column are not significantly different by LSD.

**Table 5:** Fibrous root length (cm) and tuber dry weight (g) as affected by different concentrations of gibberellic acid (GA_3_) and humic acid (HA).

Treatments	60 DAS		90 DAS		120 DAS	
	TFL	TDW	TFL	TDW	TFL	TDW
Control	980.30^d^	1.03^f^	1689.90^d^	3.39^e^	1044.89^a^	5.27^d^
10 mg L^−1^ GA_3_	1677.60^c^	2.16^e^	1939.70^cd^	3.75^e^	1081.27^a^	6.20^cd^
15 mg L^−1^ GA_3_	2235.80^ab^	3.31^c^	2697.80^b^	6.36^c^	1077.00^a^	7.68^b^
20 mg L^−1^ GA_3_	2452.60^a^	4.53^a^	2927.60^b^	7.10^b^	1080.80^a^	10.92^a^
5% HA	2066.40^b^	2.52^d^	2162.80^c^	5.20^d^	1064.68^a^	8.29^b^
10% HA	1038.20^d^	1.28^f^	1896.40^cd^	3.41^e^	1018.19^a^	7.22^bc^
15% HA	2076.70^b^	4.13^ab^	3441.30^a^	8.90^a^	1092.41^a^	11.51^a^

Prob. > *F*	219.03	0.32	285.81	0.49	44.30	1.45

DAS, TFL and TDW indicate days after sprouting, total fibrous root length (cm) and tuber dry weight (g), respectively. Means with the same letter in a same column are not significantly different by LSD.

**Table 6:** Morphological characteristics of tubers as affected by different treatments.

Treatments	Number of tubers	Average tuber length (cm)	Tuber diameter (mm)
Control	12.67^c^	12.80^a^	5.01^a^
10 mg L^−1^ GA_3_	13.67^c^	13.57^a^	5.19^a^
15 mg L^−1^ GA_3_	16.33^b^	9.18^b^	5.25^a^
20 mg L^−1^ GA_3_	19.74^a^	8.54^b^	5.65^a^
5% HA	14.00^c^	12.34^a^	5.30^a^
10% HA	9.72^d^	12.61^a^	5.18^a^
15% HA	21.46^a^	9.83^b^	5.84^a^

Prob. > *F*	1.50	1.43	0.62

Means with the same letter in a same column are not significantly different by LSD.

**Table 7:** Comparison between percentage (%) in weight per bulk, yield, and cost of two treatments.

Treatment	Increase in weight/bulk (%)	Increase in yield per ha (%)	Increase in cost per ha (%)
20 mg L^−1^ GA_3_	38.0	17.0	8.1
15% HA	37.0	19.0	2.0

Note: price for making 20 mg L^−1^ GA_3_ was RM 2.20 per liter (approximately USD 0.70) and 15% HA was RM 0.45 per liter (approximately USD 0.15).
